# Cryptic breakpoint identified by whole-genome mate-pair sequencing in a rare paternally inherited complex chromosomal rearrangement

**DOI:** 10.1186/s13039-018-0384-2

**Published:** 2018-06-07

**Authors:** Constantia Aristidou, Athina Theodosiou, Andria Ketoni, Mads Bak, Mana M. Mehrjouy, Niels Tommerup, Carolina Sismani

**Affiliations:** 1 Department of Cytogenetics and Genomics, The Cyprus Institute of Neurology and Genetics, Nicosia, Cyprus; 2 The Cyprus School of Molecular Medicine, The Cyprus Institute of Neurology and Genetics, Nicosia, Cyprus; 3 Wilhelm Johannsen Centre for Functional Genome Research, Department of Cellular and Molecular Medicine, University of Copenhagen, Copenhagen, Denmark

**Keywords:** CCR, Familial, Paternal transmission, WG-MPS, Cryptic breakpoint, Reproductive problems

## Abstract

**Background:**

Precise characterization of apparently balanced complex chromosomal rearrangements in non-affected individuals is crucial as they may result in reproductive failure, recurrent miscarriages or affected offspring.

**Case presentation:**

We present a family, where the non-affected father and daughter were found, using FISH and karyotyping, to be carriers of a three-way complex chromosomal rearrangement [t(6;7;10)(q16.2;q34;q26.1), de novo in the father]. The family suffered from two stillbirths, one miscarriage, and has a son with severe intellectual disability. In the present study, the family was revisited using whole-genome mate-pair sequencing. Interestingly, whole-genome mate-pair sequencing revealed a cryptic breakpoint on derivative (der) chromosome 6 rendering the rearrangement even more complex. FISH using a chromosome (chr) 6 custom-designed probe and a chr10 control probe confirmed that the interstitial chr6 segment, created by the two chr6 breakpoints, was translocated onto der(10). Breakpoints were successfully validated with Sanger sequencing, and small imbalances as well as microhomology were identified. Finally, the complex chromosomal rearrangement breakpoints disrupted the *SIM1*, *GRIK2*, *CNTNAP2*, and *PTPRE* genes without causing any phenotype development.

**Conclusions:**

In contrast to the majority of maternally transmitted complex chromosomal rearrangement cases, our study investigated a rare case where a complex chromosomal rearrangement, which most probably resulted from a Type IV hexavalent during the pachytene stage of meiosis I, was stably transmitted from a fertile father to his non-affected daughter. Whole-genome mate-pair sequencing proved highly successful in identifying cryptic complexity, which consequently provided further insight into the meiotic segregation of chromosomes and the increased reproductive risk in individuals carrying the specific complex chromosomal rearrangement. We propose that such complex rearrangements should be characterized in detail using a combination of conventional cytogenetic and NGS-based approaches to aid in better prenatal preimplantation genetic diagnosis and counseling in couples with reproductive problems.

## Background

Complex chromosomal rearrangements (CCRs) are generally defined as structural rearrangements that involve more than two chromosome breaks resulting in exchanges of chromosomal segments [1]. The occurrence of constitutional CCRs is rare with approximately 250 cases reported so far [2, 3]. The majority of apparently balanced CCR carriers are phenotypically normal [2]. However, affected CCR carriers have been previously reported presenting with intellectual disability or other clinical phenotypes. These develop mainly through dosage-sensitive gene disruption [4], disruption of *cis*-regulatory elements, thus, affecting the expression of disease-candidate genes via long-range position effect [5, 6], presence of cryptic imbalances near the breakpoints or elsewhere in the genome [7–9], as well as unmasking of recessive variants by the CCR on the intact chromosomes [1, 10]. In addition, male infertility [11], recurrent miscarriages [12], as well as stillbirths are common reproductive problems associated with otherwise healthy couples carrying apparently balanced CCRs.

Pregnancy outcomes in CCR carriers have been investigated first by Gorski et al. [13]; the risk for miscarriages and abnormal pregnancies in couples with CCRs was estimated to be at 48.3 and 53.7%, respectively [13]. However, these are general guidelines and since most CCRs are unique in each carrier or family, it is strongly recommended that individual CCRs should be investigated separately [2]. Accurate prediction of the phenotypic outcome of each pregnancy and reproductive risk estimation is challenging in the case of CCRs because of the different malsegregation patterns and recombination events that can occur resulting in unbalanced gametes [2, 3]. In addition, the higher the complexity of a CCR (i.e. increasing number of chromosomes and breakpoints involved in a rearrangement) and the possibility of recombination events, the higher the percentage of unbalanced gamete generation and the risk for having an affected offspring [2, 3]. Therefore, precise characterization of balanced CCRs is crucial in terms of estimating a more accurate percentage for reproductive risk and abnormal pregnancies, and thus, providing better genetic counseling in couples carrying such complex rearrangements.

High resolution next generation sequencing approaches have been proven fruitful for detailed investigation of CCRs [4]. We have previously demonstrated that whole-genome mate-pair sequencing (WG-MPS) is highly efficient in accurately mapping familial apparently balanced reciprocal translocation breakpoints [14]. Moreover, our group and others have also shown that WG-MPS, often in combination with conventional methods, is a powerful tool for revealing additional complexity in CCR carriers, including chromothripsis rearrangements, that could remain undetected by using only conventional methods with lower resolution (manuscript in preparation) [15, 16].

In this study, WG-MPS was applied in order to further characterize and delineate the breakpoints of a de novo CCR involving chromosomes 6, 7, and 10 in a phenotypically normal male with reproductive failure in his family. By revealing the full complexity of the CCR, we aim to provide more precise abnormal pregnancy risk estimations and better genetic counseling in individuals carrying the specific CCR.

## Case presentation

### Case report and preliminary analyses

**Fig. 1:**
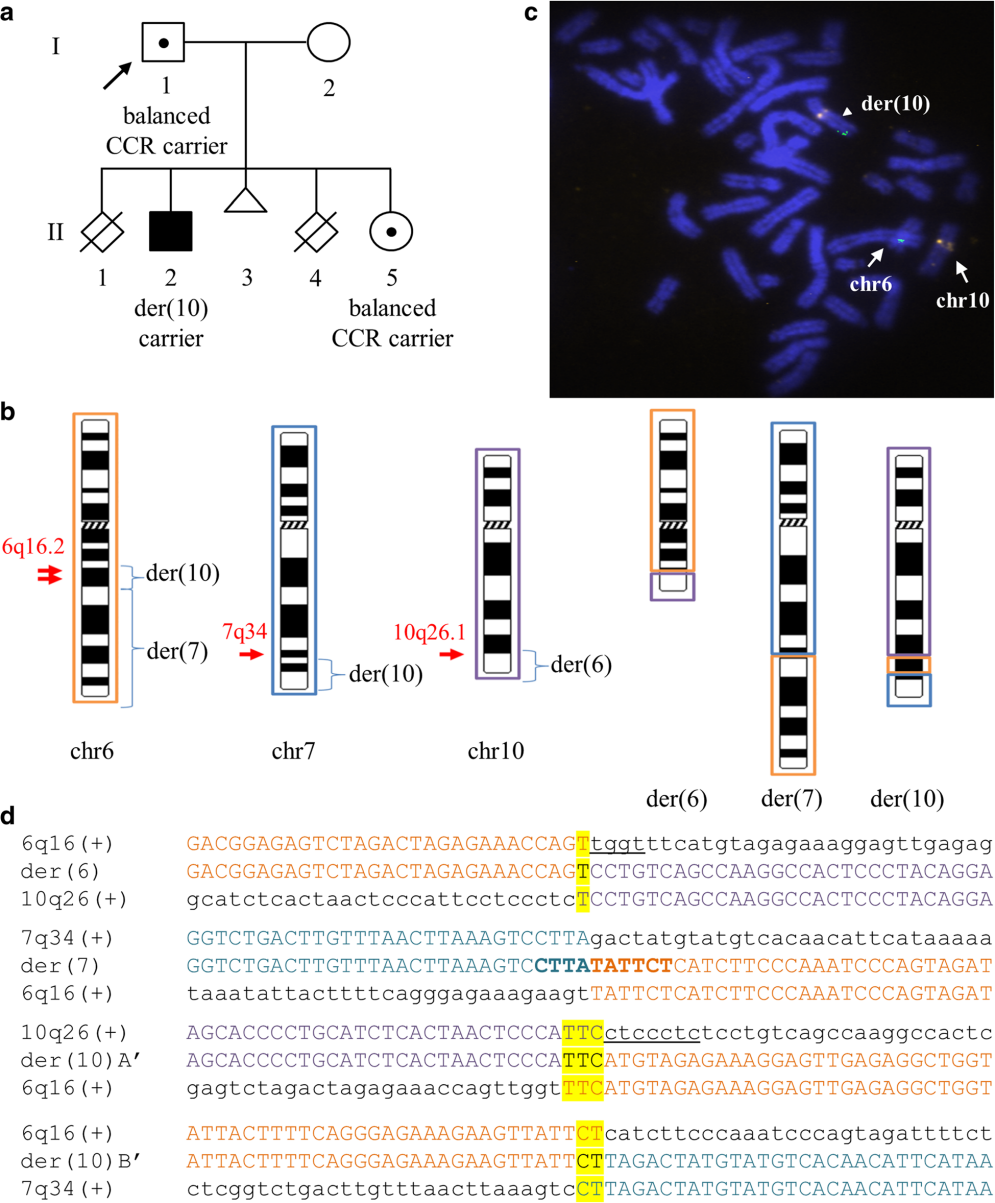
Family Pedigree, Whole-Genome Mate-Pair Sequencing and FISH Results. **a** Family pedigree depicting the non-affected father (I:1), non-affected daughter (II:5), and affected son (II:2) with severe intellectual disability. The family also suffered from two stillbirths (II:1 and II:4) and one miscarriage (II:3). **b** Ideograms displaying the normal and derivative chromosomes 6 (orange), 7 (blue) and 10 (purple) (not to scale). The approximate breakpoint positions on 6q16.2, 7q34, and 10q26.1 are indicated by arrows, and the derivative chromosomes onto which each segment is translocated are also shown. **c** FISH results using a custom-designed probe within 6q16.3 (green signal) and a control probe within 10q11.22 (orange signal) on metaphase spreads from the non-affected daughter. Both signals are visible on der(10) (arrowhead), and as expected, a green and an orange signal were seen on normal chromosomes 6 and 10 (arrows), respectively. The same results were also observed in the non-affected father (not shown). **d** CCR breakpoint sequences as identified by WG-MPS and verified by Sanger sequencing. Derivative chromosome sequences (middle line) and matching reference sequences are in capital letters. Microhomology is highlighted, deleted sequences around the breakpoints are underlined, and duplicated sequences are in bold letters

A family was referred to the Department of Cytogenetics and Genomics, as they suffered from two still births (II:1 and II:4) and one miscarriage (II:3). They also have a son with severe intellectual disability (II:2) and a non-affected daughter (II:5) (Fig. 1a).

Initial chromosomal analysis performed elsewhere using conventional G-banding at the 550-band level detected a de novo chromosomally apparently balanced translocation (ABT) involving chromosomes (chr) 6 and 7 in the non-affected father (I:1) [46,XY,t(6;7)(q16;q34)], while a normal karyotype [46,XX] was detected in the non-affected mother (I:2). Subsequent Fluorescence In-Situ Hybridization (FISH) analyses by Patsalis et al. [17] revealed cryptic complexity and the involvement of chr10 as well in the rearrangement. At that time, the karyotype of the father was revised as 46,XY,t(6;7;10)(q16.2;q34;q26.1)dn. The non-affected daughter inherited the same CCR [46,XX,t(6;7;10)(q16.2;q34;q26.1)pat], whereas the affected son inherited only der(10) and normal chromosomes 6 and 7 from the father [46,XY,der(10)t(6;7;10)(q16;q34;q26)pat], resulting in a partial 10qter monosomy (~ 6 Mb) and 7qter trisomy (~ 11.5 Mb) [17].

### Whole-genome mate-pair sequencing

WG-MPS library preparation, using 1 μg DNA from the father and the Nextera Mate-Pair Sample Preparation kit Illumina, San Diego, CA, USA), sequencing on HiSeq2500, WG-MPS data analysis using Burrows-Wheeler Aligner-MEM [18], SVDetect [19], and Integrative Genomics Viewer [20], as well as translocation breakpoint validation with Polymerase Chain Reaction (PCR) and Sanger sequencing were done as previously described [14].

### Fluorescence In-Situ Hybridization

FISH analyses were performed, using a custom-designed FISH probe on 6q16.3 and a control probe on 10q11.2 (BlueGnome Ltd., Cambridge, United Kingdom), on fixed chromosome suspensions from the father and daughter according to the manufacturer’s protocols.

## Results

In the current study, WG-MPS in the father revealed a cryptic translocation breakpoint on chr6, thus rendering the rearrangement even more complex as compared with the three-way CCR identified from the initial karyotype and FISH analyses. In total, four translocation junctions were identified by WG-MPS; two on chr6 (~ 1.37 Mb apart from each other), one on chr7 and one on chr10 (Table 1). The interstitial segment created from the additional cryptic translocation breakpoint on chr6 was translocated on der(10) proximal to the 7q34-qter segment (Fig. 1b); this was validated with FISH using a custom designed probe within the chr6 interstitial segment and a control probe on chr10 (Fig. 1c).

**Table 1:** Complex rearrangement breakpoint junctions as delineated by whole-genome mate-pair sequencing (WG-MPS) and Sanger sequencing (SS)

Chromosomal break	Translocation junctions as predicted by WG-MPS (GRCh37/hg19)	Translocation breakpoint positions as defined by SS (GRCh37/hg19)
chr6 (1st break)	chr6:100899302-100900111 [TRANSLOC_BAL_18reads_chr10:129761169-129761668]	chr6:100899825-100899830
chr6 (2nd break)	chr6:102274568-102275034 [TRANSLOC_BAL_13reads_chr7:147888949-147890271]	chr6:102274901-102274908
chr7	chr7:147888949-147890271 [TRANSLOC_BAL_13reads_chr6:102274568-102275034]	chr7:147889469-147889474
chr10	chr10:129761169-129761668 [TRANSLOC_BAL_18reads_chr6:100899302-100900111]	chr10:129761568-129761576

After reconstructing all derivative chromosomes (Fig. 1b), breakpoints were successfully mapped to the base-pair level by Sanger sequencing in both non-affected father and daughter using the same PCR primer pairs (Fig. 1d; Table 1). Breakpoint positions, as well as microhomology and small imbalances around the breakpoints were identical in both CCR carriers (Fig. 1d; Table 1). The two der(10) breakpoint junctions were also successfully amplified and sequenced in the affected son who inherited only der(10). As expected, no PCR product was observed after amplifying der(6) and der(7) translocation breakpoint junctions in the affected son (not shown).

Each CCR breakpoint disrupted known genes; single-minded family bHLH transcription factor 1 (*SIM1*) (NM_005068.2) (intron 2) on chr6 (1st break), glutamate ionotropic receptor kainate type subunit 2 (*GRIK2*) (intron 9) (NM_021956.4) on chr6 (2nd break), contactin associated protein-like 2 (*CNTNAP2* or *CASPR2*) (intron 18) (NM_014141.5) on chr7, and protein tyrosine phosphatase, receptor type E (*PTPRE*) (intron 1) (NM_006504.5) on chr10. Finally, none of the identified translocation breakpoints occurred within or near any conserved non-coding *cis*-regulatory element regions for long-range position effects.

## Discussion

Familial CCRs tend to have fewer breakpoints and are mainly maternally transmitted via oogenesis, as in the case reported by Binsbergen et al. [21] where a three-way CCR was unstably transmitted from a non-affected mother to her affected son, while de novo CCRs tend to have more breakpoints and the majority of them are paternal in origin arising during spermatogenesis [2]. Nevertheless, a few cases of familial CCRs with paternal transmission have been documented in the past leading to unbalanced or recombinant rearrangements in the offspring [13, 22]. The fact that complex rearrangements affect spermatogenesis [13, 23] and, subsequently, infertility and subfertility often associated with male CCR carriers [1, 2], are plausible etiologies underlying this limited paternal transmission of CCRs.

In the current study, we present a rare case of familial CCR stably transmitted from a non-affected father to his non-affected daughter. Previous reports suggested that the specific CCR involved a single breakpoint on each *q*-arm of the participating chromosomes 6, 7, and 10, and the reciprocal exchange of the terminal segments created [17]. However, WG-MPS utilized in the present study allowed accurate reconstruction of the derivative chromosomes, and interestingly, revealed a cryptic translocation breakpoint on chr6 (Fig. 1b). The interstitial chr6 segment translocated onto der(10) (Fig. 1b) was confirmed by FISH (Fig. 1c), validating the power of WG-MPS in delineating rearrangement complexity. Because of the relatively short translocation breakpoint junctions suggested by WG-MPS (~ ≤ 1 kb), breakpoint mapping to the base-pair level was feasible with the use of a single primer pair spanning each breakpoint junction. Breakpoint locations and molecular “signatures” were identical in all non-affected members, thus confirming that the CCR was stably transmitted from the father to his daughter, while malsegregation of the derivative chromosomes probably led to the inheritance of the unbalanced rearrangement in the son.

Even though a single known protein-coding gene was disrupted by each of the four CCR breakpoints in our study, such heterozygous disruption was phenotypically inconsequential. *SIM1* haploinsufficiency has been associated with obesity in previous mice studies [24] and reports of patients carrying *SIM1* loss-of-function variants [25–27] or chromosomal abnormalities in the *SIM1* gene region [28, 29]. However, the pathogenic impact of *SIM1* disruption is inconsistent as *SIM1* variants have also been reported, similar to the cases presented here, in lean, control individuals [25–27, 30]. Such phenotypic discordances can be partly explained by the presence of more complex rearrangements affecting, sometimes in addition to *SIM1*, other genes associated with obesity and neurodevelopmental phenotypes in affected individuals [30, 31] or identification of rearrangements that may protect against obesity in non-obese individuals [30]. It has also been suggested that complex gene-gene or gene-environment interactions may additionally influence the degree of the obesity phenotype penetrance [27]. Homozygous loss-of-function *GRIK2* variants have been reported in patients with moderate to severe non-syndromic autosomal recessive mental retardation [32]. In addition, two de novo, heterozygous microdeletions in *cis* position on chromosome 6q16.1q16.2 and 6q16.3 disrupting, among others, the *PRDM13* and *GRIK2* genes have been reported in a patient with intellectual disability and autism; however, the authors concluded that functional interaction between both disrupted genes most probably underlies phenotype presentation [33]. Heterozygous *CNTNAP2* disruptions reported in affected individuals presenting with autism spectrum disorder [34, 35] or Gilles de la Tourette syndrome and Obsessive Compulsive Disorder [36] were mostly located at the proximal part of the *CNTNAP2* gene [34, 36] and/or were unbalanced [36], or the rearrangements were even more complex affecting other disease-candidate genes as well [34, 35]. Homozygous *CNTNAP2* variants have also been reported in affected patients with cortical dysplasia-focal epilepsy syndrome [37] or CASPR2 deficiency syndrome characterized by intellectual disability, autistic features and language impairment [38]. These examples are in contrast to those reported in healthy individuals where the *CNTNAP2* gene is disrupted at more distal sites: within intron 11 by a t(7;15) translocation as reported by Belloso et al. [39], and within intron 18 by the CCR reported here. Thus, results from the present study support the suggestion that smaller and more distal *CNTNAP2* disruptions may be phenotypically inconsequential [35]. Furthermore, we cannot exclude the possibility that the proximal and distal *CNTNAP2* gene fragments are expressed as functional fusion genes with the distal and proximal *GRIK2* gene fragments on der(7) and der(10), respectively, as both genes are expressed on the plus strand. Taken together, the common genetic phenomena of incomplete penetrance and variable phenotypic expression of *SIM1* and *CNTNAP2* disruptions, the recessive mode of inheritance of *GRIK2*-related phenotypes, the possibility of functional fusion gene generation, as well as the absence of additional chromosomal rearrangements affecting clinically relevant genes within the same pathways as the genes disrupted here by the CCR may explain the absence of specific clinical phenotypes in the father and daughter reported in the present study.

**Fig. 2:**
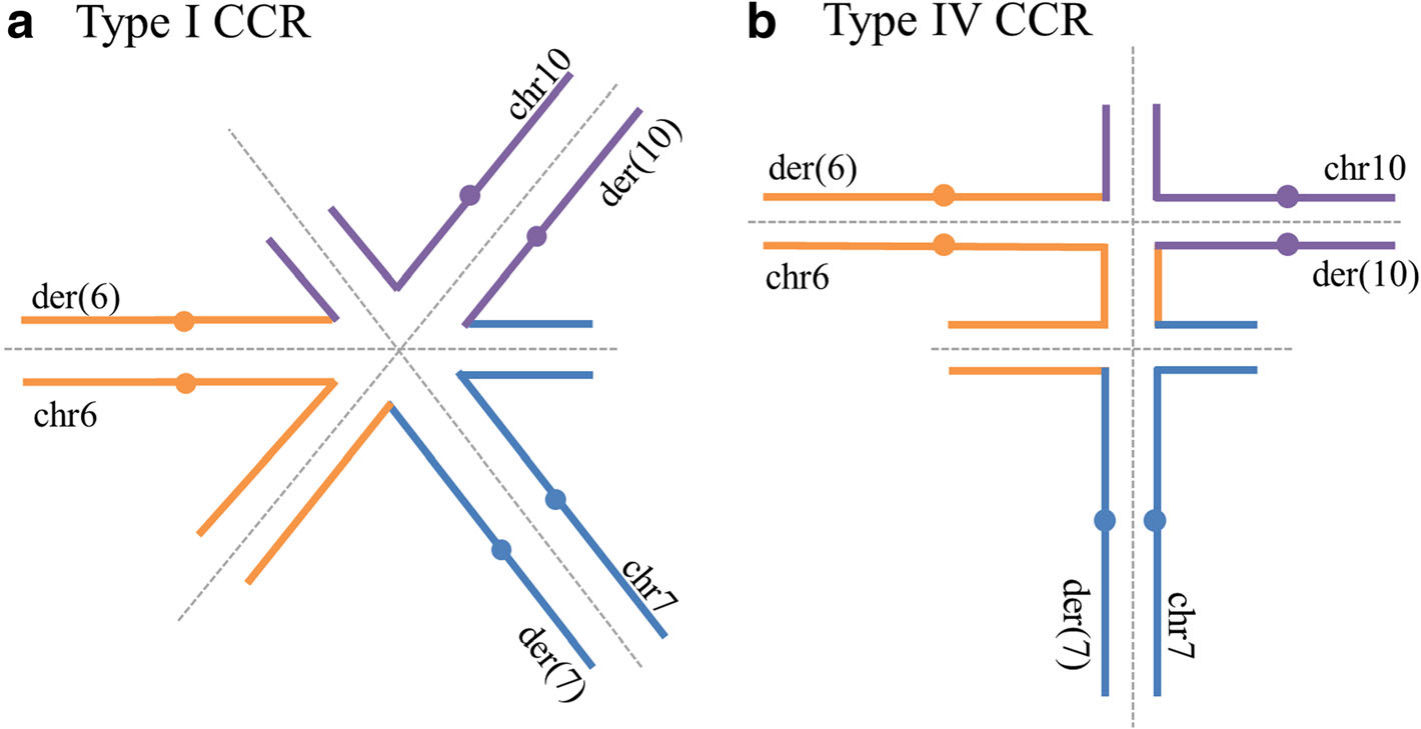
Type I and Type IV CCR Hexavalent Configurations. Different hexavalent configurations in case of: **a** type I CCR, as determined by previous analyses, and **b** type IV CCR, as refined by whole-genome mate-pair sequencing in the current study. The additional breakpoint as well as possible recombination at the “middle segment” in type IV CCR increases the percentage of unbalanced gametes, and subsequently, reproductive risk. Genetic material from chromosomes 6, 7, and 10 are illustrated in orange, blue, and purple lines, respectively

With the use of WG-MPS and the identification of a cryptic breakpoint, the CCR in this study was refined from a type I CCR (number of breaks = number of chromosomes) to a type IV CCR (number of breaks>number of chromosomes and there is a “middle segment”), based on the classification system proposed by Madan [3]. Specifically, the CCR here involves three chromosomes and four breakpoints, while the “middle segment” is the interstitial chr6 fragment translocated onto der(10). Type I and type IV CCRs align in different hexavalent configurations during the pachytene stage of meiosis I (Fig. 2). The cryptic chr6 breakpoint combined with possible recombination at the “middle segment” can produce new rearrangements and result in higher reproductive risk, increased unbalanced gamete production, and consequently, affected offspring [3]. More specifically, it has been estimated that there is an additional ~ 3.5% risk per breakpoint, whereas there is a ~ 35% possibility for a recombination event to occur in type IV CCRs, and recombination may generally result in both unbalanced and balanced gametes [3, 21, 22].

Recent technological advances in next generation sequencing focus the investigation of chromosomal rearrangements, including CCRs, towards higher-resolution breakpoint mapping and precise interpretation at the gene level [4]. While such approaches are highly successful in characterizing chromosomal rearrangements in detail and may reveal additional levels of complexity [15], such as in the present family reported here, conventional karyotype and molecular cytogenetic analyses remain nonetheless pivotal systemic strategies to investigate three-dimensional genome topology changes [5, 6]. Thus, multi-level analysis using a combination of NGS and conventional cytogenetic techniques should be used instead as a holistic approach for the investigation of CCRs to gain a more complete understanding of the overall genomic system. In general, this would aid in monitoring genome instability, which can often be further induced by the use of assisted reproductive technologies, in infertile couples carrying chromosomal rearrangements [40].

## Conclusions

In conclusion, the present study investigates a rare case where a phenotypically inconsequential CCR is stably transmitted from a fertile male carrier to his daughter. To the best of our knowledge, this is the first report of an apparently balanced CCR involving chromosomes 6, 7 and 10, and additional complexity discovered through WG-MPS in a family with reproductive problems. Together with previous findings, our study highlights the strength of WG-MPS as a methodology for accurate detection and characterization of CCRs. Even though the exact percentage of unbalanced gametes and reproductive risk cannot be fully determined in couples carrying CCRs, detailed characterization of individual CCRs using a combination of conventional cytogenetic and NGS-based methods remains nonetheless highly important to reveal their full complexity, as well as provide better prenatal preimplantation genetic diagnosis and genetic counseling.

## Abbreviations

ABT: Apparently balanced translocation
CCR: Complex chromosomal rearrangement
chr: Chromosome
der: Derivative
FISH: Fluorescence In-Situ Hybridization
NGS: Next generation sequencing
PCR: Polymerase Chain Reaction
WG-MPS: Whole-genome mate-pair sequencing
